# A Recombinant Porcine Reproductive and Respiratory Syndrome Virus Stably Expressing DsRed Protein Based on Bacterial Artificial Chromosome System

**DOI:** 10.3389/fmicb.2022.839845

**Published:** 2022-01-21

**Authors:** Na Li, Yiyi Zhang, Lunguang Yao, Yunpeng Shi, Qin Zhao, Baicheng Huang, Yani Sun

**Affiliations:** ^1^Key Laboratory of Ecological Security, Collaborative Innovation Centre of Water Security for Water Source Region of Mid-line of South-to-North Diversion Project of Henan Province, Henan Provincial Engineering and Technology Center of Health Products for Livestock and Poultry, School of Life Sciences and Agricultural Engineering, Nanyang Normal University, Nanyang, China; ^2^Shijiazhuang Customs (Huanghua Port), Cangzhou, China; ^3^College of Veterinary Medicine, Northwest A&F University, Yangling, China; ^4^National Research Center for Veterinary Medicine, Luoyang, China

**Keywords:** HP-PRRSV, DsRed, bacterial artificial chromosome, transcription regulatory sequence, reporter

## Abstract

Recombinant viruses possessing reporter proteins as tools are widely applied in investigating viral biology because of the convenience for observation. Previously, we generated a recombinant pathogenic porcine reproductive and respiratory syndrome virus (PRRSV) with enhanced green fluorescent protein (EGFP) reporter for monitoring virus spread and screening of neutralizing antibodies. PRRSV with different kinds of reporters can support more application scenarios. Here, we described a new genetically stable infectious clones of a highly pathogenic PRRSV (HP-PRRSV) harboring the DsRed (a red fluorescent protein isolated from the coral *Discosoma*) gene. In the recombinant infectious clone, the transcription regulatory sequence 2 (TRS2) of PRRSV was inserted between the open reading frame 7 (ORF7) and 3′UTR to drive the transcription of DsRed gene, which makes it a separate transcription unit in the viral genome. Using the bacterial artificial chromosome (BAC) system and cytomegalovirus (CMV) promoter, the recombinant HP-PRRSV with the DsRed insertion was successfully rescued and showed similar growth and replication patterns compared with the wild-type virus in the MARC-145 cells. In addition, the DsRed protein was stably expressed in the recombinant virus for at least 10 passages with consistent fluorescence intensity and density. Using the recombinant HP-PRRSV with DsRed protein, the virus tracking in MARC-145 was observed by live-cell imaging. Meanwhile, quantification of the DsRed fluorescence positive cells by flow cytometry provides an alternative to standard methods for testing the level of PRRSV infection. This recombinant PRRSV with DsRed fluorescence protein expression could be a useful tool for fundamental research on the viral biology and shows the new design for stable expression of foreign genes in PRRSV.

## Introduction

Porcine reproductive and respiratory syndrome (PRRS) is a widespread disease affecting domestic pigs, first reported in North America and Europe in the late 1980s (Wensvoort et al., 1991; Albina, 1997). The symptoms of PRRS include reproductive failure, pneumonia, and increased susceptibility to secondary bacterial infection. The causative agent of PRRS is the PRRS virus (PRRSV), which is in the *Arteriviridae* family of the order *Nidovirales*. PRRSV is an enveloped, single-stranded positive-sense RNA virus of approximately 15 kb in length that contains 9 open reading frames (ORFs) (Fang and Snijder, 2010). Two distinct viral genotypes of PRRSV are North American type and European type, only shared the genome identity by approximately 60%. In the early 2000s, the outbreak of highly pathogenic PRRS (HP-PRRS) inflicted serious economic losses, which characterized by high-grade fever with increased mortality in pigs of all ages (Tian et al., 2007). The modified attenuated and killed vaccines have been wildly used in the field (Charerntantanakul, 2012). However, there is no remarkably effective vaccine for controlling PRRSV infection in pigs.

In the past decade, reverse genetics technology was applied to many RNA viruses to generate recombinant mutant viruses for virology and biology studies (Racaniello and Baltimore, 1981; Bernstein et al., 1986; Gonzalez et al., 2002; Almazan et al., 2006; St-Jean et al., 2006; Park et al., 2012; Amarilla et al., 2021; Nouda et al., 2021). Many infectious clones of different PRRSV strains have been constructed, including North American and European type strains (Nielsen et al., 2003; Truong et al., 2004; Fang et al., 2006; Zhang et al., 2011; Sang et al., 2012; Wang et al., 2013). In various rescue systems, the bacterial artificial chromosomes (BAC) have the advantages of large antigen-capacity, high fidelity in replication, and the easier procedures of manipulation, which was applied both in DNA (Messerle et al., 1997; Borst et al., 1999; Suter et al., 1999; Kanda et al., 2004) and RNA virus (Almazan et al., 2000; Almazan et al., 2006; Wang et al., 2013). The reverse genetics technology with fluorescence protein insertion is a common strategy for recombinant virus labeling, which gives an intuitive convenience for virus observation by fluorescence microscopy or flow cytometer. The application of fluorescence proteins in virus labeling mainly focuses on the enhanced green fluorescent protein (EGFP). Previously, we generated a recombinant PRRSV with EGFP expression, and the transcription regulatory sequence for ORF6 (TRS6) was inserted between the ORF7 and 3′UTR to drive the transcription of the EGFP (Wang et al., 2013). The results showed that the recombinant PRRSV with EGFP was a useful tool for neutralizing antibodies screening. However, to study the real-time interaction of various proteins, it is necessary to achieve multi-fluorescence labeling for live-cell imaging (Wolff et al., 2006; Strickfaden et al., 2010; Behrens et al., 2017).

In this study, to produce a differentiated recombinant PRRSV for fluorescence capture, we developed an HP-PRRS virus (HP-PRRSV) infectious clone with a red fluorescence protein of DsRed (a red fluorescent protein isolated from the coral *Discosoma*) based on BAC system. The DsRed gene was inserted between the ORF7 gene and the 3′UTR region of the PRRSV genome and transcripted by TRS2 (the sequence length of TRS2 is shorter than that of TRS6). The expression levels and stability of the DsRed in the rescued recombinant PRRSV were investigated, in addition, the virus tracking in MARC-145 and the quantification of the DsRed fluorescence positive cells by flow cytometry were also studied.

## Materials and Methods

### Plasmid and Cell Lines

The PRRSV infectious clone plasmid pBAC-SD16*^FL^*-AM containing the whole viral genome derived from the HP-PRRSV strain (SD16 strain, GenBank: JX087437) was constructed based on the results on previous descriptions (Wang et al., 2013). MARC-145 cell line, derived from the embryonic African green monkey kidney cell line (MA-104), was cultured in the Dulbecco’s modified Eagle’s medium (DMEM) supplemented with 10% fetal bovine serum (FBS) (HyClone, South Logan, UT, United States) at 37°C in a 5% CO_2_ incubator.

### Plasmid Construction With DsRed Gene Insertion

**FIGURE 1:**
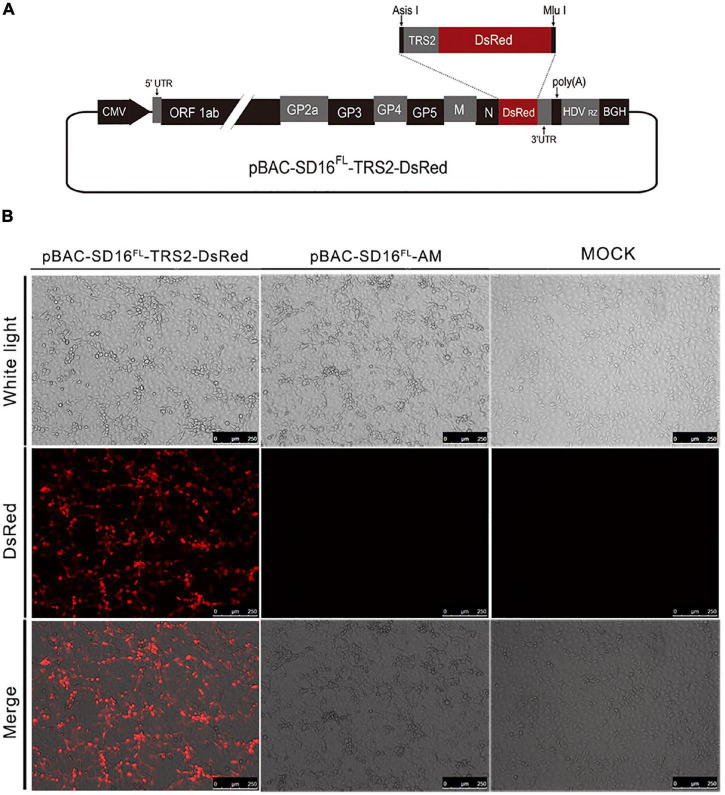
Plasmid construction of pBAC-SD16*^FL^*-TRS2-DsRed and DsRed expression in MARC-145 cells after transfection. **(A)** The DsRed gene was flanked by the transcription regulatory sequence 2 (TRS2) at the 5′end was inserted into plasmid pBAC-SD16*^FL^*-AM to produce plasmid pBAC-SD16*^FL^*-TRS2- DsRed using sites of *Asi*SI and *Mlu*I; **(B)** MARC-145 cells transfected with pBAC-SD16*^FL^*-TRS2-DsRed or pBAC-SD16*^FL^*-AM in a 6-well plate, after 48 h post-transfection, the fluorescence pictures in the live cells were captured by fluorescence microscopy.

The plasmid with DsRed gene insertion was constructed as previously described (Wang et al., 2013). Briefly, the DsRed gene was amplified from pDsRed-Express-N1 Vector (Clontech, CA, United States) with primers 5′-GCGATCGC *TTGAACCAACTTTAGGCCTGAATTGAA*ATGGCCTCCTCCG AGGC-3′ (the *Asi*SI site is underlined and TRS2 sequence is in italics), and 5′-CAGCCCACGACGCGTCGCTACAGGAA CAGGTGGTG-3′ (the *Mlu*I site is underlined) by polymerase chain reaction (PCR). The amplified product was inserted into the pEasy™-blunt simple cloning vector to generate plasmids pEasy-TRS2-DsRed. After sequencing, the positive plasmids containing TRS2-DsRed genes and pBAC-SD16*^FL^*-AM were digested with *Asi*SI and *Mlu*I, and then the digested products were ligated to generate plasmid pBAC-SD16*^FL^*-TRS2-DsRed. The design of the recombinant PRRSV infection clone is shown in Figure 1A. The recombinant plasmid was identified by sequencing. Finally, the positive plasmid was extracted with the QIAfilter Plasmid Midi Kit (QIAGEN, Hilden, Germany) and transfected into MARC-145 cells.

### Transfection and Rescue of Recombinant Viruses

The recombinant PRRSV carrying DsRed was rescued according to the method in previous report (Wang et al., 2013). Briefly, MARC-145 cells in a 6-well plate (80% confluent) were transfected with the plasmids pBAC-SD16*^FL^*-TRS2-DsRed using Attractene Transfection Reagent (QIAGEN, Hilden, Germany) following the manufacturers’ instructions. After 2 days of incubation at 37°C and 5% CO_2_, the cytopathic effect (CPE) in cells were checked daily, and the fluorescence was captured when the CPE was found. In addition, the culture supernatants from the transfected cells were used to infect MARC-145 cells to propagate the rescued virus. The rescued viruses were confirmed by sequencing. The rescued virus was named rSD16/TRS2-DsRed.

### Immunofluorescence Assays

MARC-145 cells were cultured on glass coverslips in a 24-well plate and were infected with SD16 or rSD16/TRS2-DsRed (3rd passage) at a multiplicity of infection (MOI) of 0.01 for 48 h. Cells were fixed with 4% formaldehyde for 30 min at room temperature (RT) and permeabilized with 0.5% Triton X-100 in phosphate-buffered saline (PBS) for 10 min. Then, the cells were incubated with monoclonal antibody (mAb) against PRRSV nucleocapsid (N) protein (6D10) for 1 h at room temperature as previously described (Li et al., 2016). After that, the cells were still incubated with Alexa Fluor^®^ 488 AffiniPure Goat Anti-Mouse IgG (Jackson, West Grove, PA, United States) for 1 h at RT. Finally, the cells were stained with 4-6-diamidino-2-phenylindole (DAPI) for 5 min at RT. Images were taken by Leica microscope. Mock-infected MARC-145 cells were used as controls.

### Western Blotting

MARC-145 cells were infected with SD16 or rSD16/TRS2-DsRed (3rd passage) at an MOI of 0.01. When 60% of the cells showed CPE, the infected cells were collected and lysed. The cellular proteins were separated by SDS-PAGE and transferred on a polyvinylidene difluoride (PVDF) membrane. The membrane was incubated with anti-α-Tubulin antibody (Sigma-Aldrich, MO, United States), mouse anti-DsRed mAb (Santa Cruz Biotechnology, United States) and 6D10 mAb overnight at 4°C. And then, it was incubated with peroxidase-conjugated goat anti-mouse IgG (Jackson, PA, United States) for 1 h at RT. Immuno-stained proteins were visualized using ECL Western Blotting Substrate (Pierce, CA, United States). Cellular proteins from mock-infected MARC-145 cells were used as controls.

### Imaging of Porcine Reproductive and Respiratory Syndrome Virus With DsRed Expression

MARC-145 cells were grown on a 35 mm cell culture dish to a density of 50–70% confluence and then infected with rSD16/TRS2-DsRed (MOI: 0.1) of different passages. After 1 h post-infection (hpi), the medium was removed and replaced with 2 mL of pre-warmed DMEM containing 3% FBS. The dish was placed at 37°C with 5% CO_2_. The fluorescence of different passages of rSD16/TRS2-DsRed was captured by Leica microscope at 36 hpi. In live-cell imaging, MARC-145 cells were infected with rSD16/TRS2/DsRed at an MOI of 0.01 in a 35 mm cell culture dish. At 1 hpi, the medium was removed and replaced with 2 ml of pre-warmed DMEM containing 3% FBS. The dish was placed in the 37°C observation chamber containing 5% CO_2_ (Leica CTR-Controller 3700, Wetzlar, Germany). DsRed signals and phase-contrast images were captured with the time interval of 1 min for 3 h by a live-cell station (Leica AF6000, Wetzlar, Germany). Subsequently, images were processed into a movie of 10 frames s-1 using QuickTime Pro.

### Determination of Growth Curves for Recombinant Porcine Reproductive and Respiratory Syndrome Virus

Recombinant virus rSD16/TRS2-DsRed was propagated in MARC-145 cells with an MOI of 1.0. The growth curves of rSD16/TRS2-DsRed and parental virus SD16 were compared. Briefly, 1 h after virus adsorption, MARC-145 cells were washed three times with PBS and incubated in DMEM with 3% FBS at 37°C, 5% CO_2_. The supernatants were collected at various time points (12, 24, 36, 48, 60, and 72 hpi), and viral titers were calculated based on the TCID_50_ data (Calculate by *Reed-Muench* method).

### Polymerase Chain Reaction Analysis of Viral RNA Isolation and Reverse Transcription

To determine the mRNA production of the DsRed gene in different passages, the viral RNA of rSD16/TRS2-DsRed (passages 1, 3, 5, 7, and 9) was extracted from supernatants of culture medium with the RNAiso Plus (Takara, Dalian, China) and then reverse-transcribed into cDNAs with M-MLV reverse transcriptase (Takara, Dalian, China). The target PCR fragments were amplified using the primer pairs of 5′-ATACTGTGCGCCTGATCCGC-3′ (located in the 3′ end of N protein) and 5′- TCGCCAATTAAACTTTACCCCCACA-3′ (located in the 5′ end of 3′UTR region).

## Results

### Rescue of rSD16/Transcription Regulatory Sequence 2-DsRed

A vector pBAC-SD16*^FL^*-AM with *Asi*SI and *Mlu*I sites between the ORF7 gene and the 3′UTR region of the viral genome was used to construct PRRSV infectious clone with DsRed. The DsRed gene was flanked by the unique *Asi*SI and *Mlu*I sites for its insertion into the plasmid pBAC-SD16*^FL^*-AM, and its expression was driven by the transcription-regulating sequence of gene GP2a (TRS2) (Figure 1A). An apparent DsRed fluorescence was observed in the MARC-145 cells after transfection with pBAC-SD16*^FL^*-TRS2-DsRed (Figure 1B), which means the successful expression of indictor protein.

### Replication Properties of Recombinant Viruses

**FIGURE 2:**
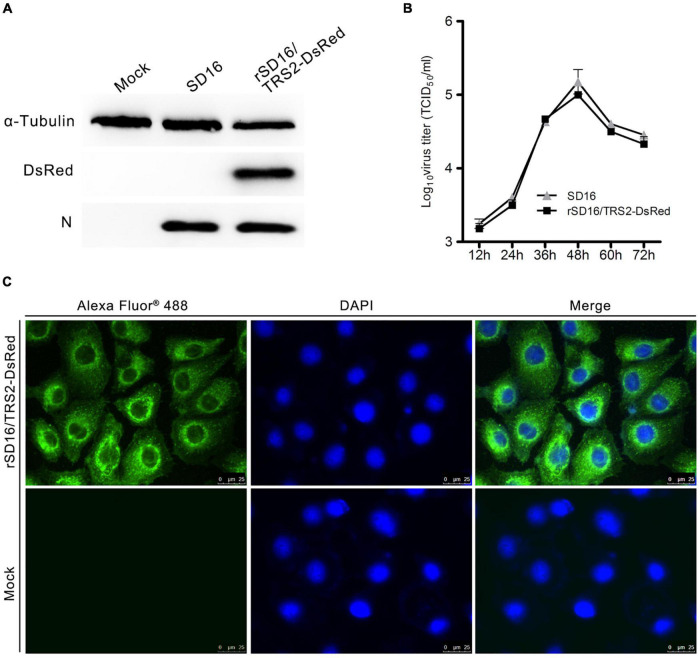
DsRed protein expression and growth curve of rSD16/TRS2-DsRed. **(A)** After being infected with SD16 or rSD16/TRS2-DsRed (MOI of 0.1) for 36 h, the MARC-145 cells were lysed for the western blot analysis of PRRSV protein and DsRed expression, the MARC-145 cells infected with SD16 and normal MARC-145 cells were taken as the control; **(B)** MARC-145 cells were infected with SD16 or rSD16/TRS2-DsRed at the MOI of 1.0, and the progeny virus produced in the culture medium was measured at a series of times from 12 to 72 hpi by the method of TCID_50_ detection, and all the data were expressed as the mean ± standard deviation (SD); **(C)** after infected with rSD16/TRS2-DsRed for 48 h, the MARC-145 cells were fixed for the immunofluorescence analysis using the monoclonal antibody of PRRSV N protein, the normal MARC-145 cells were the control.

The expression of DsRed and PRRSV N protein in MARC-145 cells infected with rSD16/TRS2-DsRed were identified by western blot using anti-DsRed and anti-PRRSV N protein mAbs as primary antibodies. As shown in Figure 2A, DsRed and PRRSV N protein were detected in rSD16/TRS2-DsRed infected cells, and DsRed was not detected in SD16-infected or normal MARC-145 cells. PRRSV N protein was also detected in virus-infected cells by immunofluorescence assays but not in the normal cells (Figure 2C). The replication ability of the recombinant virus with DsRed insertion was also compared by growth curves of SD16 and rSD16/TRS2-DsRed in MARC-145 cells. The supernatant of the culture medium of rSD16/TRS2-DsRed and the parental virus were collected at 12, 24, 36, 48, and 60 hpi, respectively. The viral titers were examined at the indicated time points. As shown in Figure 2B, the growth kinetics of recombinant PRRSV rSD16/TRS2-DsRed was not significantly different from the parental virus, and the titers peaked at 48 hpi for both viruses.

### Stability of DsRed Expression in Recombinant Viruses

**FIGURE 3:**
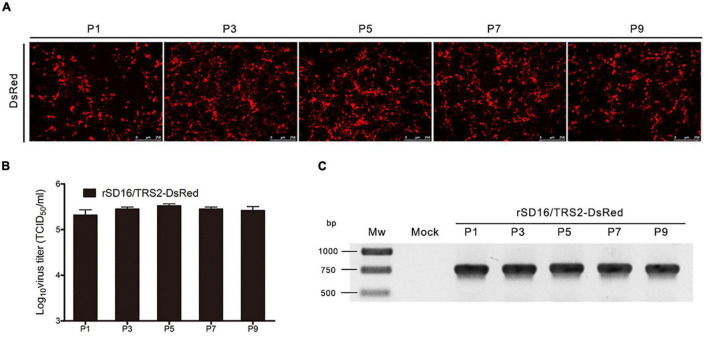
Stable expression of DsRed in different passages of rSD16/TRS2-DsRed. **(A)** In different passages, fluorescence pictures of the recombinant virus were captured at 48 hpi by fluorescence microscopy; **(B)** titers of the recombinant virus were detected at different passages by TCID_50_ method, all the tests were repeated three times; **(C)** mRNA production of DsRed gene at different passages of the recombinant virus were detected by reverse transcription PCR analysis.

The recombinant viruses were passaged in MARC-145 cells for at least 10 passages to investigate the stability of DsRed expression. As visualized by fluorescence microscopy, positive cells exhibited a sustained red fluorescence at different passages (Figure 3A). The viral titers of the recombinant virus in different passages were also detected by the TCID_50_ method. As shown in Figure 3B, no significant changes of the viral titers were found in different passages. Furthermore, the presence of the DsRed gene in the viral genome was identified by RT-PCR. As shown in Figure 3C, all fragments were consistent with the expected length of about 750 bp. In addition, the PCR products were also sequenced, and the results showed no mutations in the DsRed gene (data not shown).

### Intercellular Spread of rSD16/Transcription Regulatory Sequence 2/DsRed

Live-cell imaging was performed in recombinant virus-infected MARC-145 cells to determine whether the rSD16/TRS2/DsRed could be used as a reporter virus. As shown in Supplementary Movie 1, the DsRed fluorescent in the recombinant virus-infected cells could be observed at approximately 24 hpi, and the fluorescence is distributed evenly in the cells. We also found that the DsRed fluorescence could be transported along intercellular connecting nanotube to neighboring cells. These results suggested that the recombinant PRRSV rSD16/TRS2/DsRed can be used as a reporter virus to analyze the biological characterizations of PRRSV.

### DsRed as an Indicator of Recombinant Virus Infection

**FIGURE 4:**
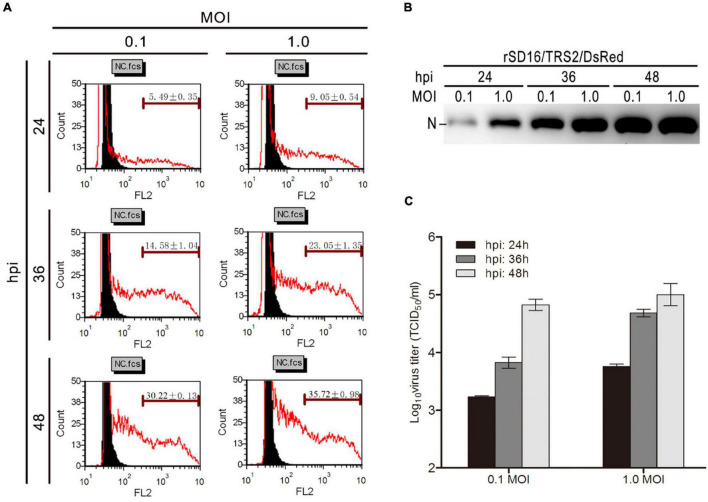
DsRed as an indicator of recombinant PRRSV infection. **(A)** Different concentrations of DsRed in virus-infected cells detected by flow cytometry; **(B)** western blot analysis to detect PRRSV N proteins expression after infection with rSD16/TRS2/DsRed; **(C)** titers of culture supernatant were detected using the TCID_50_ method, and all the tests were repeated three times.

To validate the correlation between the DsRed and the viral infection, the DsRed fluorescence intensity from the infected virus was measured by flow cytometry. As shown in Figure 4A, a gradient manner of red fluorescence positive cells ratio in MARC-145 cells infected with rSD16/TRS2/DsRed in different hpi. The ratio of positive cells ranged from 5.49% at hpi of 24 (MOI: 0.1) to 35.72% at hpi of 48 (MOI: 1.0), indicating a high ratio of DsRed positive cells when the culture time was prolonged. Western blot analysis of PRRSV N protein and TCID_50_ detection of culture supernatant were performed to verify whether the ratio of fluorescence positive cells is related to the virus infection. As shown in Figures 4B,C, the gradient manner is similar to the flow cytometry results of different hpi.

## Discussion

One of the most important achievements in RNA virus research is the development of reverse genetics systems, which makes the possibility of the virus genetic manipulation (Ishibashi et al., 2017). Reverse genetics technology has been extensively utilized to explore various aspects of virus infection, including infection mechanisms, virulence, pathogenesis, immune responses, transmission characteristics, vaccine development, and antiviral screening tests (Hoenen et al., 2011). There are two strategies to construct a reverse genetics system for PRRSV depending on whether viral RNA or cDNA is used for transfection (Chaudhari and Vu, 2020). It has been demonstrated that the DNA-based transfection system produces 10–100-fold higher viral titers than the RNA-based transfection system (Huang et al., 2009). The BAC system is based on the *Escherichia coli* (*E. coli*) F factor, in which replication is strictly controlled in *E. coli*. In BAC system, structural stability of inserted DNA in the host on the higher degree at the lower number of copies per cell (one or two per cell), beside the capability of maintaining large fragments (Shizuya et al., 1992), which applied in the rescue of many kinds of virus, comprising virus belongs to *Coronaviridae* (Almazan et al., 2000, 2006; Wang et al., 2013) and *Herpesviridae* (Messerle et al., 1997; Borst et al., 1999; Suter et al., 1999; Kanda et al., 2004; Li et al., 2011). Based on this system, we revealed that the ∼15.4 kb fragment of the PRRSV viral genome was inserted in the BAC plasmid for transfection more conveniently without steps like *in vitro* transcription.

Genetically recombinant viruses which express green and red fluorescent proteins are universally used as tools for studying replication and spread of diverse RNA viruses in living cells or animals (Kanai et al., 2019). Although PRRSV with EGFP has been generated (Wang et al., 2013), this is the first report of a recombinant HP-PRRSV expressing red fluorescence. DsRed, derived from the coral *Discosoma*, has an orange-red fluorescence and can form a stable tetramer, which is detectable by laser-based confocal microscopes and flow cytometers with an excitation at 568 nm (Hawley et al., 2001). It was possible to simultaneously measure the efficiency and fluorescence intensity of the transfected live cells. Compared with EGFP, DsRed has shown its tremendous advantages, including bright red fluorescence and high resistance against photo-bleaching (Garcia-Parajo et al., 2001). Recombinant viruses expressing DsRed proteins represent a better option to combine with genetically modified GFP-expressing cell lines or animals. Their reduced auto-fluorescence background captures the dynamics of viral infection and replication (Chiem et al., 2021). DsRed expression was used for the high-throughput hepatotoxicity test in drug screening and biomonitoring environmental toxicants in zebrafish (Zhang et al., 2014), the investigation of the host and tumor cell compartments (Jacobsen et al., 2013), and identification of MHC I-restricted epitope in mouse (Davey et al., 2013). In the cells, which were used in high-throughput scale detection of allergic sensitization (Wang et al., 2014), *in vivo* imaging of metastatic disease (Yu et al., 2012), genotoxicity, and oxidative stress assessment (Hendriks et al., 2011). As showed in this study, the DsRed fluorescence in the recombinant HP-PRRSV was observed in the MARC-145 cells with CPE (Figure 1).

Meanwhile, the growth kinetics of recombinant PRRSV rSD16/TRS2-DsRed showed no significant difference compared with that of the parental virus (Figure 2). The DsRed fluorescence intensity and the viral titers were stable at different passaged-recombinant PRRSV (Figure 3). Using the rSD16/TRS2/DsRed as a tool, the virus tracking in MARC-145 was observed by live-cell imaging (Supplementary Movie 1). Quantification of the DsRed fluorescence positive cells was carried out by using flow cytometry, which shows it is a quick and reliable way to detect the level of PRRSV infection (Figure 4).

In conclusion, a recombinant HP-PRRSV carrying the DsRed gene as a separate transcription unit based on the BAC system was successfully rescued. The recombinant viruses can be useful for studying PRRSV transcription and replication by visualization. In addition, the strategy using a TRS2-derived foreign gene expression provides a new method for expressing foreign genes in the PRRSV genome. In the studies of PRRSV visualization, to ensure the fluorescent proteins not interfering with the natural infection state of the virus is still the challenge, which was just as reported in the study of PRV (Yang et al., 2020). In the future, CRISPR technology to achieve viral nucleic acid labeling can explain the biological characteristics of the virus that are closest to the real states.

## Data Availability Statement

The original contributions presented in the study are included in the article/Supplementary Material, further inquiries can be directed to the corresponding authors.

## Author Contributions

BH, YaS, and QZ conceived and designed the experiments. NL, BH, and YZ performed the experiments. NL, LY, and YuS analyzed the data of experiments and drafted the manuscript. All authors contributed to the article and approved the submitted version.

## Conflict of Interest

YuS was employed by Shijiazhuang Customs (Huanghua Port). The remaining authors declare that the research was conducted in the absence of any commercial or financial relationships that could be construed as a potential conflict of interest.

## Publisher’s Note

All claims expressed in this article are solely those of the authors and do not necessarily represent those of their affiliated organizations, or those of the publisher, the editors and the reviewers. Any product that may be evaluated in this article, or claim that may be made by its manufacturer, is not guaranteed or endorsed by the publisher.

## Supplementary Material

Supplementary Movie 1 Live imaging of rSD16/TRS2/DsRed observed by live-cell microscopy. MARC-145 cells were infected with rSD16/TRS2/DsRed at an MOI of 0.01 in a 35 mm cell culture dish. DsRed fluorescence signals were acquired with the time interval of 1 min for 3 h using a live-cell station, as described in Materials and methods. Images were processed into a movie of six frames s^–1^ by using QuickTime Pro. Scale bar, 50 μm.
